# Greener Strategy for Lupanine Purification from Lupin Bean Wastewaters Using a Molecularly Imprinted Polymer

**DOI:** 10.1021/acsami.2c02053

**Published:** 2022-04-14

**Authors:** Teresa Esteves, Flávio A. Ferreira, Ana Teresa Mota, Ángel Sánchez-González, Adrià Gil, Késsia H. S. Andrade, Carlos A. M. Afonso, Frederico Castelo Ferreira

**Affiliations:** † iBB—Institute for Bioengineering and Biosciences and Department of Bioengineering, Instituto Superior Técnico, Universidade de Lisboa, Av. Rovisco Pais, Lisboa 1049-001, Portugal; ‡ Associate Laboratory i4HB—Institute for Health and Bioeconomy at Instituto Superior Técnico, Universidade de Lisboa, Av. Rovisco Pais, Lisboa 1049-001, Portugal; § Centro de Química e Bioquímica and BioISI—Biosystems and Integrative Sciences Institute, DQB, Faculdade de Ciências, Universidade de Lisboa, Campo Grande, Lisboa 1749-016, Portugal; ∥ Research Institute for Medicine (iMED, ULisboa); Faculty of Pharmacy, Universidade de Lisboa, Avenida Prof. Gama Pinto, Lisboa 1649-003, Portugal

**Keywords:** molecularly imprinted polymer, computational chemistry, lupin bean debittering, lupanine, added-value compound recovery from wastewaters

## Abstract

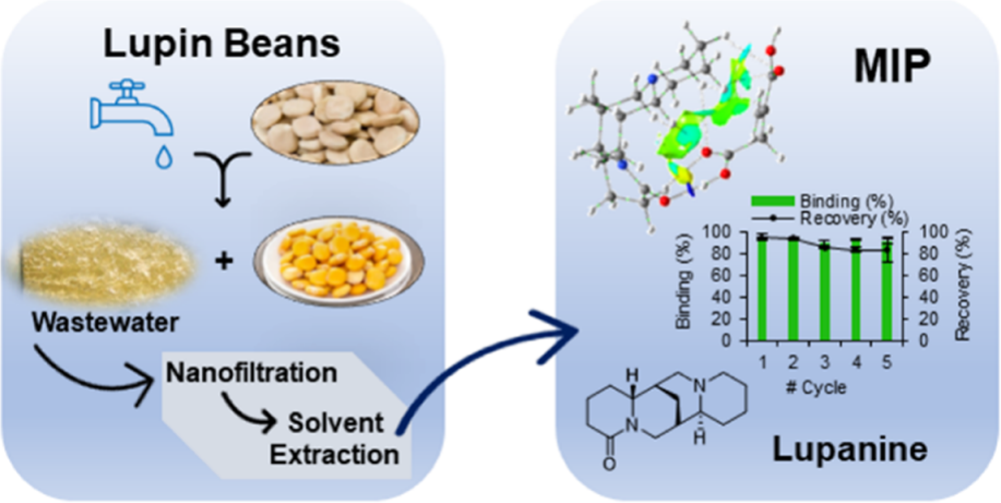

Lupanine is an alkaloid used in the pharma industry as a building block or precursor in the synthesis of sparteine and also explored for drug synthesis in the pharma industry as a chiral selector. This alkaloid is found in lupin bean processing wastewaters originated from the debittering process to make these beans edible. In this work, a computational chemistry approach was taken to design molecularly imprinted polymers (MIPs) selecting itaconic acid, a biobased building block, as a functional monomer that can provide higher affinities for lupanine. **MIP-1** was prepared using lupanine as the template, itaconic acid as a functional monomer, and ethylene glycol dimethacrylate as a cross-linker by bulk polymerization. Lupanine was concentrated from lupin bean wastewater by nanofiltration, extracted with ethyl acetate, and purified using the synthesized MIP. **MIP-1** was able to selectively recognize lupanine and improve the purity of lupanine from 78 to 88%, with 82% recovery of the alkaloid. These results show the potential application of this strategy to render the industrial process more sustainable.

## 1 Introduction

**Figure 1:**
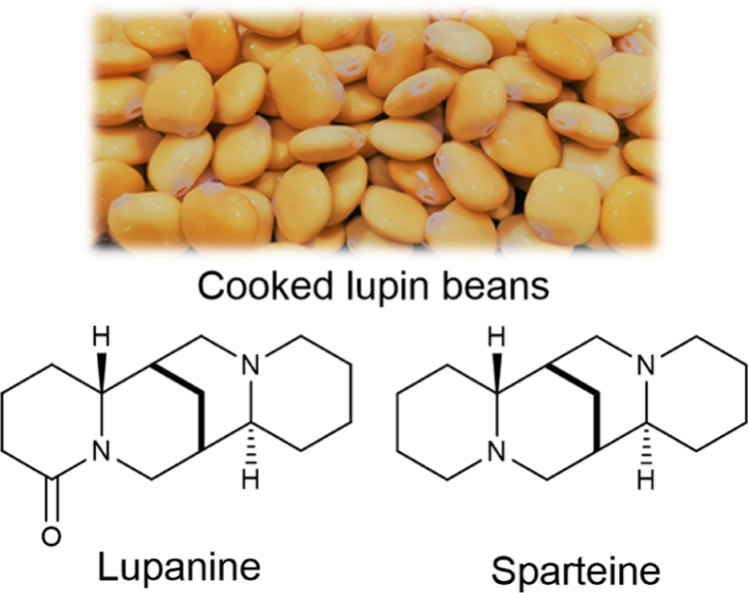
Lupin beans and lupanine and sparteine chemical structures.

Lupin beans (Figure 1) are highly nutritious seeds, low in fats and sugars, and have a high fiber content, being used as a snack or as a protein source in food products (pastry products, bread, mayonnaise, and hamburgers).^1−4^ The seeds present a bitter taste due to the presence of a toxic alkaloid, lupanine (Figure 1).^5,6^ For the seeds to become edible, the alkaloid must be removed using several m^3^ of fresh water, usually in the continuous or batch mode. This process is called debittering and comprises several stages: hydration, cooking, and thorough washing of the seeds. At the end of the process, while edible seeds are obtained, a high volume of wastewater with high contents of lupanine is also generated. Lupanine toxic effects are well-documented,^7−9^ and regulated values are followed for incorporation in food products.^10−13^ However, lupanine is a versatile building block with potential application in the pharmaceutical industry, being assessed for the treatment of type 2 diabetes and as the precursor of sparteine (Figure 1), which is a recognized chiral selector.^14^ Furthermore, its complex chemical structure makes its synthesis quite challenging, therefore making its recovery of utmost importance from the wastewaters of lupin bean processing and making this an added-value compound of great interest.

Although several studies exist in the literature concerning alternative methods for the debittering process of lupin beans,^15^ the industrial food-grade process continues to rely on the water-intensive processing of the beans.^16^ However, only a few attempts have been made to isolate lupanine from the generated wastewaters. Carmali et al. explored reverse osmosis coupled to solid–liquid extraction with ethyl ether to isolate 18.5% lupanine from the wastewaters with 90% purity.^17^ In our group, we have explored nanofiltration (NF) coupled to solvent extraction (SE) to recover 95% lupanine with 78% purity.^18^ At the same time, we also explored commercial resins to preferentially adsorb lupanine from the wastewaters and perform its recovery. XAD-16 was the best performing resin, achieving 75% isolated lupanine with only 48% purity after the NF stage. Still, the need of this adsorption stage has been overcome as a relatively higher purity-grade lupanine was achieved with SE.

Nevertheless, to purify the lupanine isolated from the wastewater, further selective separation is then required because of the coexistence of compounds with similar functional groups, for example, from the alkaloid family, such as lupanine and sparteine. Because commercial resins are not designed to perform in organic solvents and show high affinity for compounds with similar chemical functionalities, we decided to design an adsorber with high affinity for lupanine, which could be explored to recover this alkaloid from the ethyl acetate (EtOAc) fraction, ultimately improving its purity. Molecular imprinting relies on the creation of binding pockets, complementary to the chemical functionalities of the target compound, the template, in a polymeric matrix.^19,20^ This is achieved by the selection of suitable functional monomers able to establish strong interactions with the template molecule, forming a monomer–template complex that becomes entrapped in the polymeric matrix. At the end of the polymerization step, the template is removed, originating the molecularly imprinted polymer (MIP) with high affinity for the template molecule. Because we aim to recover lupanine at the end of the process, these interactions must be of reversible nature, relying mainly on electrostatic and H-bond interactions.^21^ Due to their low cost, ease of synthesis, high chemical stability, and high affinity for the target molecule, MIPs have a wide range of potential applications such as sensors, in drug delivery, in separations, for sample treatment, and for chiral resolution, for example.^22,23^

Presently, there is an urge for sustainable polymer production, using building blocks derived from green sources. Itaconic acid (IA) falls in this category, being obtained by bio-fermentation of lignocellulosic biomass.^24,25^ Its chemical functionalities, two carboxylic acid groups and one vinyl group, make it a versatile bio-based platform for the synthesis of other chemical compounds of interest or polymeric structures for wide applications such as in degradable resins, anti-microbial peptides, nanoparticles for cell imaging, stationary phases, and drug delivery.^26−30^

The novelty of this work has two vectors: material and the process based. From a material perspective, it is the first time ever that a MIP is developed successfully for lupanine isolation and successfully applied in real process streams. The strategy to develop such MIP and characterization is also, in our opinion, worth consideration as (i) **MIP-1** is made of IA, a monomer relatively unexplored for MIPs production, and due to IA bioproduction, it is indeed an advantage in the development of greener MIP synthesis and applications and (ii) mathematical modeling is used to reveal the nature of interactions of MIPs with lupanine, gaining knowledge on the specific preferential conformations of lupanine in the MIP pocket, which can inspire different approaches on MIP development.

From the process perspective, the bio-based polymer here described allows the isolation of an alkaloid from a very complex matrix, wastewater, by simple solvent extraction followed by a simple polishing step with MIP. This strategy also presents the versatility to be further explored in other food processing industries, originating alkaloid-rich waste streams such as, α-solanine and α-chaconine from potato peel waste, for example.

In this report, MIPs were prepared by bulk polymerization, using lupanine as the template and ethylene glycol dimethacrylate (EGDMA) as a cross-linker in the presence of AIBN as the initiator. Several functional monomers were assessed. Molecular modeling studies supported the choice of IA as the functional monomer, and complete characterization of the best performing adsorber was performed including isotherm binding, kinetic, and reutilization studies. The recovery of lupanine is assessed using industrial wastewaters, showing the potential applicability of the material developed here.

## 2 Materials and Methods

### 2.1 Materials

Methacrylic acid (MAA, 99.5%), IA (99%), and ethylene glycol dimethylacrylate (EGDMA, 98%) were purchased from Acros Organics. Methyl methacrylate (MMA, 99%), styrene (St, 99%), *N*-isopropylacrylamide (NIPAM, 97%), and CDCl_3_ (99.8%) used to record ^1^H NMR spectra were purchased from Sigma-Aldrich. Azobisisobutyronitrile (AIBN, 98%) was purchased from Fluka. Absolute ethanol (EtOH, 99.8%), hydrochloric acid (HCl) 37% aqueous solution, EtOAc (99.8%), dichloromethane (DCM, 99.8%), methanol (MeOH, 99.8%), and acetonitrile (MeCN, 99.9%) of HPLC grade were purchased from Fisher Scientific. Methyl *tert*-butyl ether (MTBE, 99.9%) was purchased from Lab Scan. Potassium hydroxide (KOH, 85.8%) pellets and sodium hydrogenphosphate (Na_2_HPO_4_, 99%) were purchased from Panreac. 1,3,5-Trimethoxybenzene 99% was purchased from Alfa Aesar. Soxhlet thimbles were purchased from Merck. Lupanine, with 82% purity, determined by ^1^H NMR, was obtained following a published procedure.^31^

### 2.2 Wastewater

Lupin bean wastewater was kindly provided by Tremoceira M. Ferreira Bastos Lda. (Portugal) and corresponded to the cooking stage of the industrial debittering stage. This fraction was also representative of a retentate obtained after concentration of the entire wastewater generated after processing one batch of lupin beans by NF, presenting the highest concentration of lupanine, as reported previously.^18^ The wastewater considered for the reported studies presented 3.32 g/L lupanine and 32.88 gO_2_/L COD.

### 2.3 Apparatus and Analysis

Lupanine was quantified using a Hitachi LaChrom HPLC system, at room temperature, with UV detection at 220 nm, using a reverse-phase Kinetex EVO C18 100 Å column (5 μm, 250 mm × 4.6 mm, Phenomenex). The HPLC method was isocratic for 25 min, at 1 mL/min with the mobile phase of 15% MeCN and 85% Na_2_HPO_4_ (1.8 g/L) buffer adjusted with NaOH to pH 10.5, with 20 μL injection volume. Lupanine samples, obtained in different organic solvents, were left at room temperature until complete solvent evaporation. Afterward, the residue was dissolved in water, basified with KOH until pH 13–13.5, centrifuged using a 1–15P microcentrifuge (Sigma) at 10,000 rpm for 3 min, and filtered with nylon syringe filters (13 mm diameter and 0.22 μm pore size, Tecnocroma). Chromatograms of pure samples, wastewater, and recovery assays can be found in Figures S1–S3.

The specific surface area and pore diameter of the polymeric particles were determined by nitrogen adsorption according to the BET method. An accelerated surface area and porosimetry system (ASAP 2010 Micromeritics) was used under nitrogen flow.

FTIR spectra were recorded in a FTIR-ATR, Spectrum 2 (PerkinElmer) system in the 400–4000 cm^–1^ range, using 2 cm^–1^ resolution.

Visualization of the morphology of the polymeric particles was performed using scanning electron microscopy (SEM) on a FEG-SEM system (field emission gun scanning electron microscopy) from JEOL, model JSM-7001F, with an accelerating voltage set to 20 kV. Samples were mounted on aluminum stubs using carbon tape and were gold-/palladium-coated on a Southbay Technologies, model Polaron E–5100 system.

^1^H NMR spectra were obtained on a Bruker spectrometer MX300 operating at 300 MHz. Chemical oxygen demand (COD) was measured following a method described in the literature.^32^

### 2.4 Computational Details

To take into account different conformations for the interaction between lupanine and the functional monomers, docking was carried out by means of Hex 8.0.0 software.^33−35^ Default parameters of the docking control panel were used with the exception of (1) the correlation type, which was changed to shape + electro to take into account the surface shape and the electrostatic interactions and (2) the post-processing, where we perform OPLS minimization. To group the similar obtained systems, clusterization of the structures was also performed with default parameters. Subsequently, for the 10 most stable conformations, the geometries were optimized without any constraint at the M06-2X/6-311++G(d,p) level with Gaussian 09.^36^ The M06-2X functional^37^ has been proved useful and accurate for organic systems where weak non-covalent interactions play an important role.^38−41^

The optimized functional monomer–lupanine structures were characterized as minima because all frequencies were positive. For the most stable structures, QTAIM analysis^42^ along with the non-covalent interaction (NCI) index^43^ analyses was carried out by using AIMAll software^44^ to describe the nature of the weak interactions found in the systems. Such analyses have been proven useful to describe weak interactions in previous studies.^38,45,46^ Moreover, for a whole description of the interaction energies between lupanine and the considered functional monomers, the energy decomposition analysis (EDA)^47,48^ was carried to know the contribution of the different energy terms in the interaction.

Following the Kitaura and Morokuma scheme,^48^ the interaction energy (Δ*E*_int_) between two fragments (lupanine and a given monomer) can be considered as a contribution of the polarization terms and charge transfer in the so-called orbital contribution (Δ*E*_orb_), the dispersion contribution (Δ*E*_disp_), and the electrostatic contribution (Δ*E*_elstat_); all of these are attractive terms, whereas the so-called Pauli contribution (Δ*E*_Pauli_) is a repulsive term. ADF^49−51^ software has been employed to perform the EDA at the B3LYP-D3/TZP level.^52−55^

### 2.5 Preparation of Polymers

The molecularly imprinted polymers (MIPs) were prepared by bulk polymerization at the 1:4:20 ratio of the template/monomer/cross-linker (Table 1). In the synthesis of the non-imprinted polymers (NIPs), the template was absent from the reaction mixture. The selected functional monomer and the template (lupanine, 0.1 mmol) were added to a glass pressure tube containing DCM (750 μL). The resulting mixture was stirred for 5 min at room temperature. The cross-linker EGDMA and the initiator AIBN (1% w/w of total monomer weight) were added, the polymerization mixture was purged with a stream of nitrogen for 10 min at room temperature, and the tube was closed and placed at 40 °C, in a water bath, overnight with magnetic stirring. After this, the temperature was increased at 5 °C/20 min up to 65 °C, and the tube remained at this temperature for 4 h for reaction completion. The tube was then opened, and the polymer was gently crushed in a mortar.

**Table 1:**
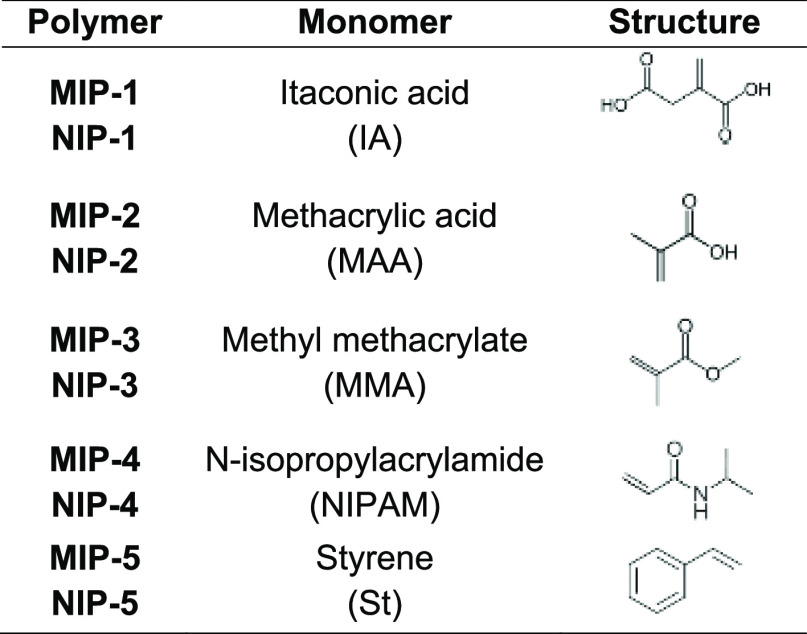
Polymers Synthesised Using Lupanine as the Template and Structure of the Functional Monomers

For template removal, the polymers prepared with IA and MAA were washed for 48 h in a Soxhlet apparatus with 0.1 M HCl in MeOH and further 24 h with MeOH for acid removal. The polymers prepared with MMA were washed in a Soxhlet apparatus for 48 h with DCM. The polymers prepared with St and NIPAM were successively washed four times in a glass beaker with a 0.1 M HCl solution in MeOH for 3 min each, with stirring and decantation, followed by three sequential washings with MeOH for acid removal. After washing, the polymers were placed in a Petri dish and dried in a Vacutherm VT 6065 vacuum oven (Thermo Scientific) at 40 °C overnight. Afterward, they were ground in a mechanical mortar, sieved (Resh stainless-steel sieves), and the fraction 38–63 μm was used for further characterization and assessment of binding performance. For the MIPs, samples of the washing solutions were evaporated to dryness and the residues were dissolved in water and processed for lupanine HPLC quantification as described in Section 2.3, confirming virtually complete template removal.

### 2.6 Batch Binding Experiments

Binding assays were performed in duplicate by adding 50 mg of each polymer and 1 mL of a solution of lupanine prepared in an organic solvent (DCM, EtOAc, MTBE, and EtOH) at 1 g/L in 2 mL Eppendorf tubes. The mixtures were allowed to stand for 24 h at room temperature at 100 rpm. After this, the mixtures were centrifuged at 10,000 rpm for 3 min in a 1–15P microcentrifuge (Sigma). The supernatant was recovered in Eppendorf tubes, and the solvent was evaporated. The obtained residues were dissolved in water and processed, as described in Section 2.3, for lupanine quantification. Lupanine binding, adsorption capacity (*q*), and the imprinting factor (IF) of the polymers were determined using eqs 1–3 respectively, where *C*_i_ (g/L) corresponds to the concentration of lupanine in the stock solution, *C*_f_ (g/L) is the final lupanine concentration, *V* (L) is the volume of solution used, and *M* (g) is the amount of the polymer.
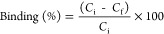
1
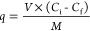
2
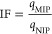
3

### 2.7 Lupanine Recovery and MIP Recyclability Experiments

Lupanine recovery and MIP regeneration were performed by addition of 1 mL of a 0.1 M HCl solution in MeOH to Eppendorf tubes with **MIP-1** after a lupanine binding experiment. The tubes stayed for 24 h in an incubation chamber (J. P. Selecta) at 55 °C, with magnetic stirring, followed by further 24 h in 1 mL of a fresh solution of 0.1 M HCl in MeOH and finally 24 h in 1 mL of MeOH. After each washing period, the tubes were centrifuged at 10,000 rpm for 3 min in a 1–15P microcentrifuge (Sigma), and the supernatants were transferred to Eppendorf tubes. After solvent evaporation, the residues were dissolved in water and processed as described in Section 2.3. for lupanine quantification. Successive lupanine binding/recovery cycles were performed, following the same steps to assess **MIP-1** reusability.

### 2.8 Adsorption Isotherm Studies

1 mL of lupanine solutions prepared in EtOAc with different initial concentration (0.1–6 g/L), was added to 50 mg of **MIP-1**. The mixtures were stirred for 24 h at room temperature at 100 rpm. After that, the suspensions were centrifuged at 10,000 rpm for 3 min in a 1–15P microcentrifuge (Sigma), and the supernatants were transferred to Eppendorf tubes, and the solvent was evaporated. The resulting residues were dissolved in water and processed as described in Section 2.3 for lupanine quantification. The amount of lupanine bound to the polymer (*q*) was calculated from eq 2.

Experimental data were fitted to the Langmuir and Freundlich isotherm models^56^ according to eqs 4 and 5, respectively, where, *C*_f_ (g/L) is the final lupanine concentration, *q*_f_ (mg/g) is the adsorption capacity for each concentration, *q*_m_ (mg/g) is the maximum amount of lupanine bound to the polymer in a monolayer for the Langmuir model, *K*_L_ (L/mg) and *K*_F_ [(mg/g)(L/mg)^1/*n*^] are equilibrium constants for the Langmuir and Freundlich models, respectively, and are related with the energy taken for adsorption, and *n* is a parameter related with the surface layer heterogeneity.
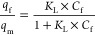
4
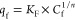
5

### 2.9 Kinetic Studies

Several mixtures were prepared with 50 mg of **MIP-1** and 1 mL of a 1 g/L solution of lupanine prepared in EtOAc and left stirring at room temperature at 100 rpm. At certain time intervals of 5, 10, 15, 30, and 45 min and 1, 2, 4, 6, 8, 24, and 27 h, the suspensions were centrifuged at 10,000 rpm for 3 min in a 1–15P microcentrifuge (Sigma), the supernatants were transferred to Eppendorf tubes, and the solvent was evaporated. The resulting residues were dissolved in water and processed for lupanine quantification, as described in Section 2.3. Experimental data were fitted to pseudo-first and pseudo-second order kinetic models^57^ according to eqs 6 and 7, respectively, where *q*_f_ and *q*_t_ (g/g) are the adsorption capacities at the final and time *t* (h), respectively, and *k*_1_ (h^–1^) and *k*_2_ [g/(g·h)] are the pseudo-first and -second-order rate constants for the models.
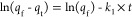
6
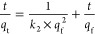
7

### 2.10 Wastewater Solvent Extraction Experiments

50 mL of lupin bean wastewater (previously basified to pH ∼ 13 with NaOH and centrifuged at 10,000 rpm for 3 min) was extracted six times with 50 mL of EtOAc. Lupanine concentration and COD were determined for each extraction stage. Lupanine extraction efficiency and purity based on COD were determined as reported previously.^18^ Lupanine purity was also determined by ^1^H NMR, for each extraction stage, using 1,3,5-trimethoxybenzene as an internal standard, in CDCl_3_.

### 2.11 Lupanine Polishing Stage Using MIP

After three consecutive extractions of lupin bean wastewater with EtOAc, as described in Section 2.10, a binding assay was performed in 10 Eppendorf tubes of 2 mL by adding 50 mg of **MIP-1** to 1 mL of the combined organic phase, with a lupanine concentration around (1.28 ± 0.08) g/L. The mixtures were stirred for 24 h at room temperature at 100 rpm. After this, they were centrifuged at 10,000 rpm for 3 min in a 1–15P microcentrifuge (Sigma). The supernatants of two Eppendorf tubes were recovered to new Eppendorf tubes, the solvent was evaporated, and the obtained residues were dissolved in water and processed, as described in Section 2.3, for lupanine quantification and binding determination using eq 1. Lupanine recovery was performed by addition of 1 mL of a 0.1 M HCl solution in MeOH to the Eppendorf tubes with **MIP-1** after the binding experiment. The tubes stayed for 24 h in an incubation chamber (J. P. Selecta) at 55 °C, with magnetic stirring, followed by further 24 h in 1 mL of a fresh solution of 0.1 M HCl in MeOH and finally 24 h in 1 mL of MeOH. After each washing period, the tubes were centrifuged at 10,000 rpm for 3 min and two Eppendorf tubes were used for quantification of lupanine by HPLC after solvent evaporation and sample treatment as described in Section 2.3. To assess lupanine purity by ^1^H NMR, the remaining supernatants were put together in a round-bottom flask, basified to pH 10–11 with a 1.23 M NaOH solution prepared in MeOH, and the solvent was evaporated. The resulting residue was washed five times with 1 mL of DCM, for lupanine extraction, filtered with a PTFE syringe filter (Tecnocroma), and transferred to a round-bottom flask. The solvent was evaporated, and lupanine purity was determined by ^1^H NMR using 1,3,5-trimethoxybenzene as an internal standard in CDCl_3_.

## 3 Results and Discussion

### 3.1 Computational Design for Functional Monomer Selection

**Figure 2:**
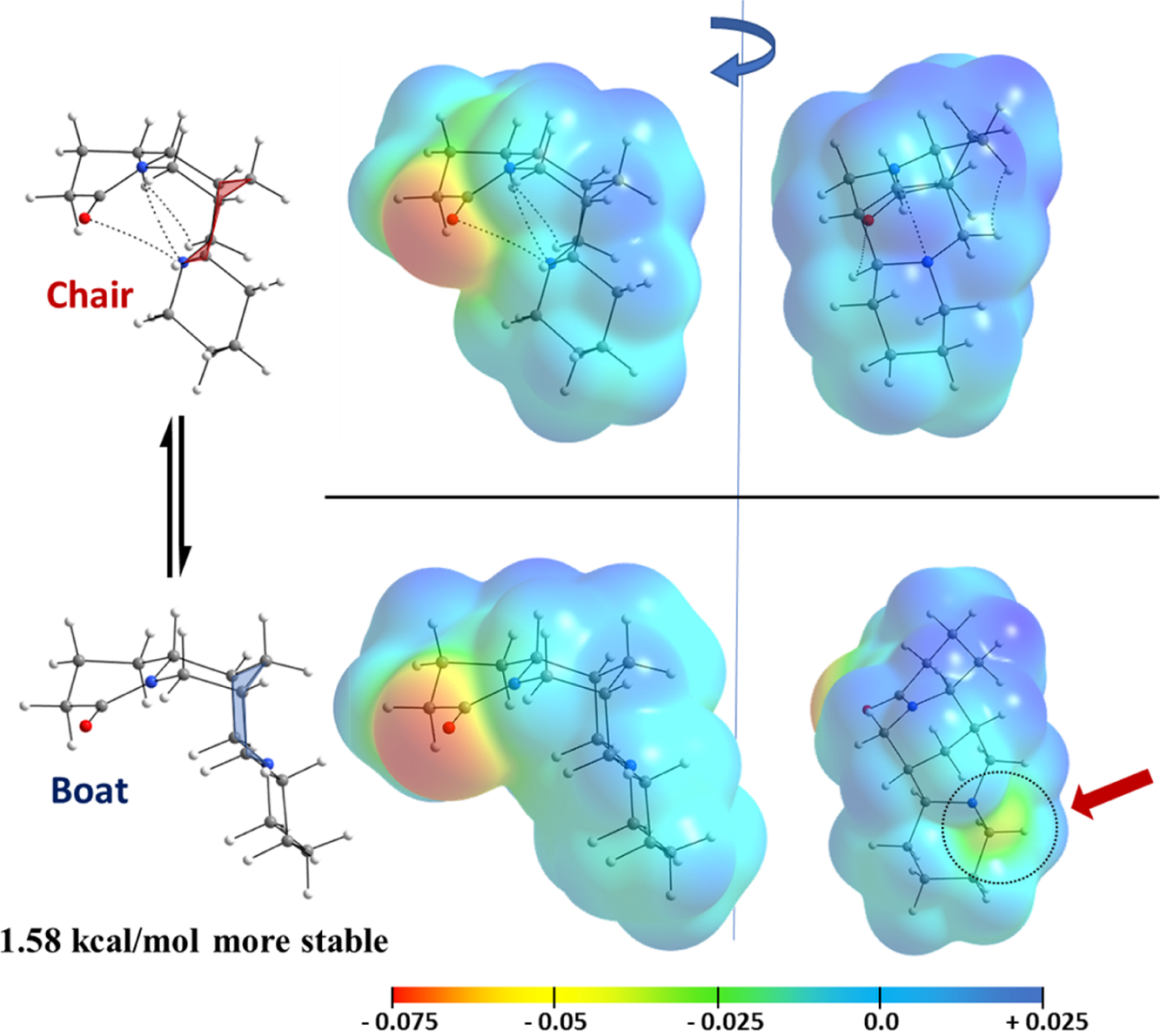
Geometric arrangements for the chair and boat conformations of lupanine with an electron density isosurface (ρ = 0.001 a.u.) mapped on the corresponding MEPs (nucleophilic regions in red and electrophilic regions in blue). Values for the electrostatic potential in a.u.

Initially, a conformational analysis for lupanine was performed. According to the ring flip of the inner ring of the molecule, two conformations are possible, in which the boat conformation is 1.58 kcal/mol more stable (Figure 2) and shows a more opened structure that avoids steric crowding. In addition, the molecular electrostatic potential maps (MEPs, Figure 2) reveal that, for the boat conformation, the electron density belonging to the N atom of the central ring is exposed (denoted with a red arrow), which allows the interaction with the solvation sphere.

**Figure 3:**
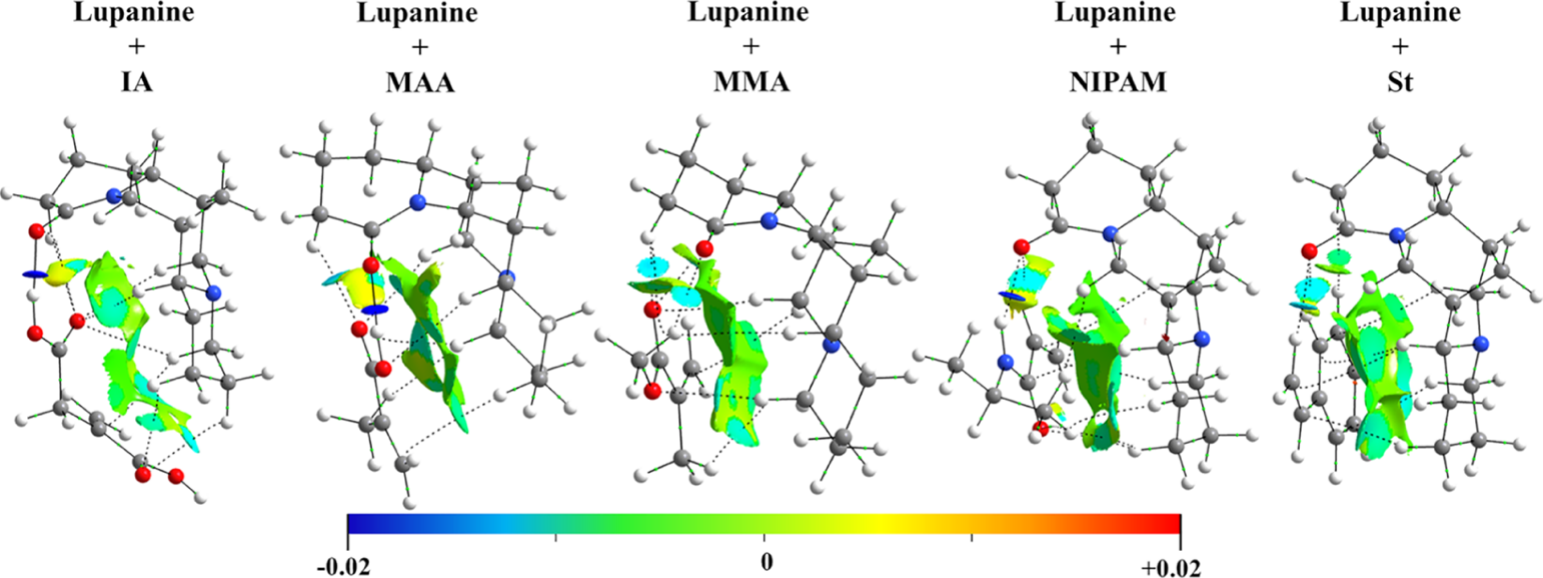
Complexes formed between lupanine and several functional monomers (in the NCI analysis, attractive forces are represented in blue and weak interactions, such as van der Waals, are represented in green).

The bonding scheme derived from the QTAIM analysis is shown in Figure 3 where the interactions between the considered functional monomer and lupanine are denoted with the bond critical points (BCPs), and the corresponding bond path is represented by dotted lines. Furthermore, analysis for the interactions is provided using the NCI index computed for the functional monomer–lupanine structure, being plotted through gradient isosurfaces in Figure 3.

We observe in Figure 3 that for all the studied functional monomer large regions represented in green (attractive interactions) appear between lupanine and the corresponding monomer. It is noteworthy that, for the system with IA, because this monomer has two carboxylic acid groups, the NCI isosurfaces reveal several areas depicted in pale blue, corresponding to the zones of the BCPs between the O atoms of IA and H atoms belonging to lupanine. Moreover, a strong stabilizing interaction is found for the H-bond formed between the −OH group of IA and the O atom of lupanine, which is represented by a lenticular dark-blue isosurface in the NCI analysis. Therefore, we can expect a strong interaction between lupanine and the IA functional monomer.

Conventional H-bonds are also shown for the system with MAA and NIPAM between the carboxylic group of MAA and the amide group of NIPAM. On the other hand, for the systems with MMA, with an ester function, and St, a benzene derivative, no conventional H-bonds are found, and these systems show weaker interactions (areas depicted in cyan). It must be highlighted that, for the system formed between lupanine and St, the smallest number for this kind of interactions is shown (only two interactions are formed).

**Figure 4:**
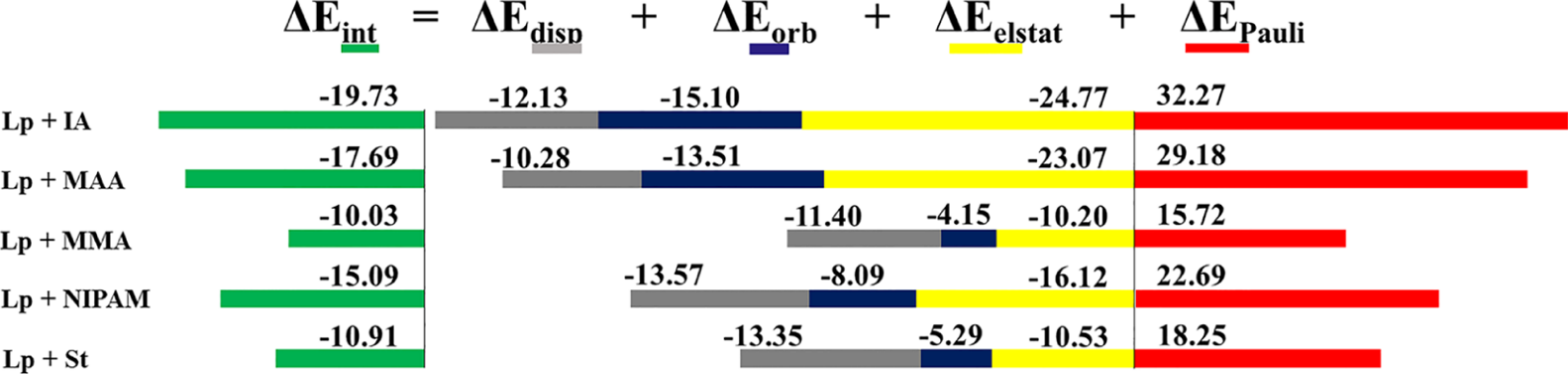
Energy contributions of the EDA represented in cumulative bar diagrams (kcal/mol), computed at the B3LYP-D3/TZP level or theory for the interaction between the template (lupanine, Lp) and the monomers.

The EDA study is shown in Figure 4 as a cumulative bar diagram. It is observed that the most favored interactions correspond to lupanine-IA and lupanine-MAA. Such systems have the most stabilized combinations despite the large repulsion energy (Δ*E*_Pauli_). This stabilization may be due to the large stabilization provided by the electrostatic contribution (Δ*E*_elstat_), being −24.77 and −23.07 kcal/mol, respectively. Such a behavior may be attributed to the capacity of IA and MAA to form strong conventional intermolecular H-bonds with lupanine as observed in Figure 3. Indeed, for these two systems, we found lenticular blue isosurfaces in the NCI plots, which correspond to OH···O conventional H-bonds. It is known that such conventional H-bonds are ruled by dispersion, but the electrostatic contribution has a major role in the nature of the interaction, as shown in the EDA.^58,59^ In addition, in these two cases (lupanine–IA and lupanine–MAA), the values of Δ*E*_orb_ are clearly higher in comparison with those of the remaining systems. We can attribute this to the charge transfer produced through the conventional H-bonds formed by the former systems, whereas for the latter systems, such conventional H-bonds do not exist, and for this reason, the values of Δ*E*_orb_ do not have such important weight.

The results provided by the EDA study are in agreement with the topological analysis of the electron density depicted above, where the most stabilized systems correspond to the structures including conventional H-bonds and OH···O interactions, which indicates that the electrostatic contribution (Δ*E*_elstat_) arising from the conventional intermolecular H-bonds is the driving force to stabilize the monomer surrounding lupanine. The order of most favored monomers to interact with lupanine is as follows: IA > MAA > NIPAM > St > MMA.

### 3.2 Lupanine Binding: Experimental Assessment

From the previous section, we observed that the interactions between lupanine and the monomers are based on non-covalent forces, essentially, H-bonds, electrostatic, hydrophobic, and van der Waals interactions, confirming that the functional monomer plays an important role in the recognition performance of a MIP. Therefore, the effect of different functional monomer chemical functionalities and interactions with the template is theoretically investigated and experimentally assessed in this section. For this purpose, the functional monomers were explored in the preparation of the MIPs for lupanine in DCM, which is an aprotic low-polarity solvent, expected to favor H-bonding and electrostatic interactions between the template and the monomers.

A MIP is efficient if there is a significant difference in performance compared to the corresponding NIP. In the case of a NIP, there is no template during polymerization, which means that the monomers will be distributed in a random way in the polymeric matrix. On the other hand, the presence of the template molecule during the synthesis of MIPs allows an ordered arrangement of the monomers around lupanine, originating binding pockets that will have a three-dimensional shape complementary to lupanine. For the synthesis, we followed a ratio of 1:4:20 for the template/monomer/cross-linker based on previous results obtained in the group and in the literature.^60,61^

For lupanine binding studies, several solvents were considered including DCM, in which the polymers were prepared. In our previous study, lupanine was eluted from XAD-16 resin using EtOH, after the lupin bean wastewater adsorption stage.^18^ In this case, EtOH would be the solvent of choice for the MIP, and therefore, it was also tested. However, optimization of lupanine isolation from wastewaters by SE revealed that EtOAc and MTBE exhibited higher lupanine recoveries and purities.^18^ Therefore, the performance of the polymers was also assessed in these two organic solvents.

**Figure 5:**
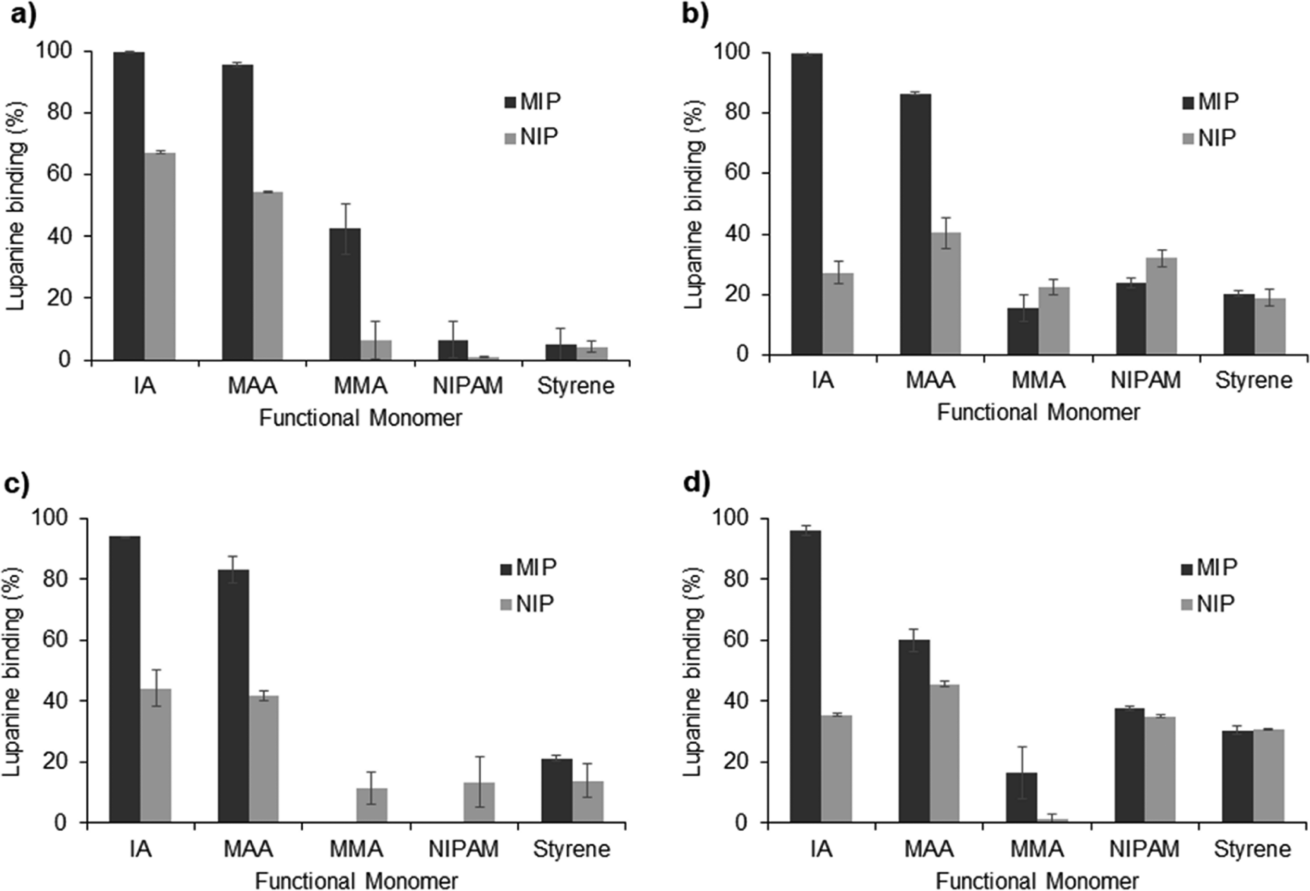
Binding of lupanine in MIP and NIP scavengers, obtained with several functional monomers, in different organic solvents: (a) DCM, (b) MTBE, (c) EtOAc, and (d) EtOH. Lupanine solutions of 1 g/L were loaded on 50 mg/mL of scavengers (*n* = 2).

From the results in Figure 5 it is possible to observe that generally, MIPs presented higher lupanine binding than the respective NIPs, as desirable. According to molecular modeling predictions, MIPs prepared with MMA, St, and NIPAM showed low lupanine binding (<40%), whereas IA and MAA originated the MIPs with higher binding for lupanine (>60%). Furthermore, for **MIP-1**, prepared with IA, even when used in a solvent that can compete with the target molecule for hydrogen bonding such as EtOH, lupanine binding was higher than 95%, being the most efficient MIP to bind lupanine, independent of the organic solvent considered. Therefore, **MIP-1** was selected for further characterization and application studies. Moreover, IA being a versatile building block produced from biological sources is an important example of MIP production with a bio-based produced monomer as an alternative to monomers produced from non-renewable resources.^24,62^

The highest imprinting factor (IF, Table S1) was obtained for MMA-based **MIP-3** (11.0) in DCM and EtOH, but corresponded to lupanine binding of only (20–40)% for the MIP and almost no recognition for the corresponding NIP. A similar trend was observed for **MIP-4** prepared with NIPAM in DCM (6.4) with a lupanine biding of only around 10% for the MIP. Besides these exceptions, the IA-based **MIP-1** showed the highest IF between 2.3 in AcOEt and 3.7 in MTBE, showing the successful binding pocket formation in the polymer matrix during polymerization.

Noteworthy, in our work, we followed a traditional bulk polymerization protocol without resorting to complicated techniques, such as using a macromolecular crowding agent or ratio optimization of different components, enabling the formation of a high-affinity MIP (**MIP-1**) for lupanine.

### 3.3 Polymer Characterization

#### 3.3.1 Morphological Characterization

The IR spectra of **MIP-1** and **NIP-1** were shown to be superimposable (Figure S4), showing that the template (lupanine) was effectively removed, after **MIP-1** synthesis. Reinforcing this observation is the absence of characteristic peaks of lupanine from **MIP-1** and **NIP-1** spectra. Furthermore, **MIP-1** and **NIP-1** spectra showed the presence of characteristic peaks attributed to the carbonyl (C=O) symmetric stretching for the cross-linker (EGDMA) and monomer (IA) in the intense band centered around 1722 cm^–1^, the carboxylate C–O stretch at 1137 cm^–1^, the O–H bands at 1448 and 948 cm^–1^, and the O–H stretch centered at 2957 cm^–1^.^29,60,63^

**Figure 6:**
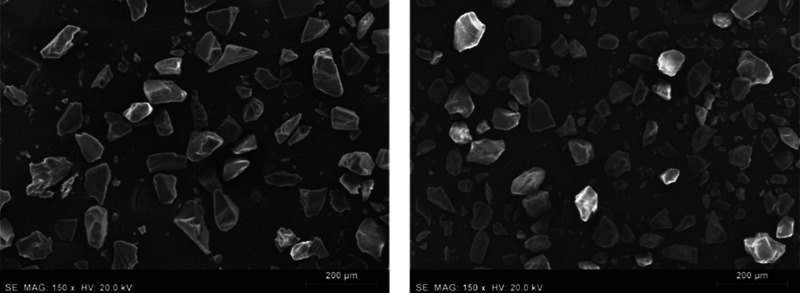
SEM images of **MIP-1** (left) and **NIP-1** (right) polymeric particles.

From SEM analysis, no significant structural difference was observed between **MIP-1** and **NIP-1** particles, showing a smooth surface (Figure 6), as in other MIPs obtained by bulk polymerization,^60^ with an average particle size around 57 nm (Figure S5). The elemental analysis also proved to be similar for **MIP-1** and **NIP-1** according to expected values (Table S2), confirming efficient template removal.

From the BET analysis (Table 2), it is possible to observe that **MIP-1** has a lower surface area than **NIP-1**. This result indicates that the higher lupanine binding observed for **MIP-1** (>95%) when compared with that for **NIP-1** (30–65%) is based on a recognition driven by selective interactions established between the template and the functional monomer during MIP synthesis, not relying on the surface area of the imprinted polymeric particles. The lower surface area for **MIP-1**, assessed using BET, may be due to the fact that IA is not completely soluble in DCM, the porogen used in MIP synthesis, chosen to favor the establishment of non-covalent interactions between the template and the monomer. It is described that, in MIP preparation, when phase separation occurs, the surface area of the resulting polymer is reduced.^64^ This effect was evident in **MIP-1** compared to the corresponding **NIP-1,** where no pre-polymerization template/monomer complex was formed.

**Table 2:** Physical Properties of **MIP-1** and **NIP-1** Obtained by the Multipoint BET Method

	BET surface area (m^2^/g)	pore volume (cm^3^/g)	average pore diameter (nm)
**MIP-1**	37.53	0.050	5.31
**NIP-1**	271.23	0.275	3.76

#### 3.3.2 Adsorption Isotherm and Kinetic Characterization

The adsorption isotherm and kinetic studies for lupanine and **MIP-1** were performed in AcOEt. Previous results showed that with this solvent, it was possible to achieve the same lupanine recovery efficiency (92–98)% and purity (77%), by SE of lupin bean-enriched NF wastewaters, using less solvent when compared to MTBE.^18^

**Figure 7:**
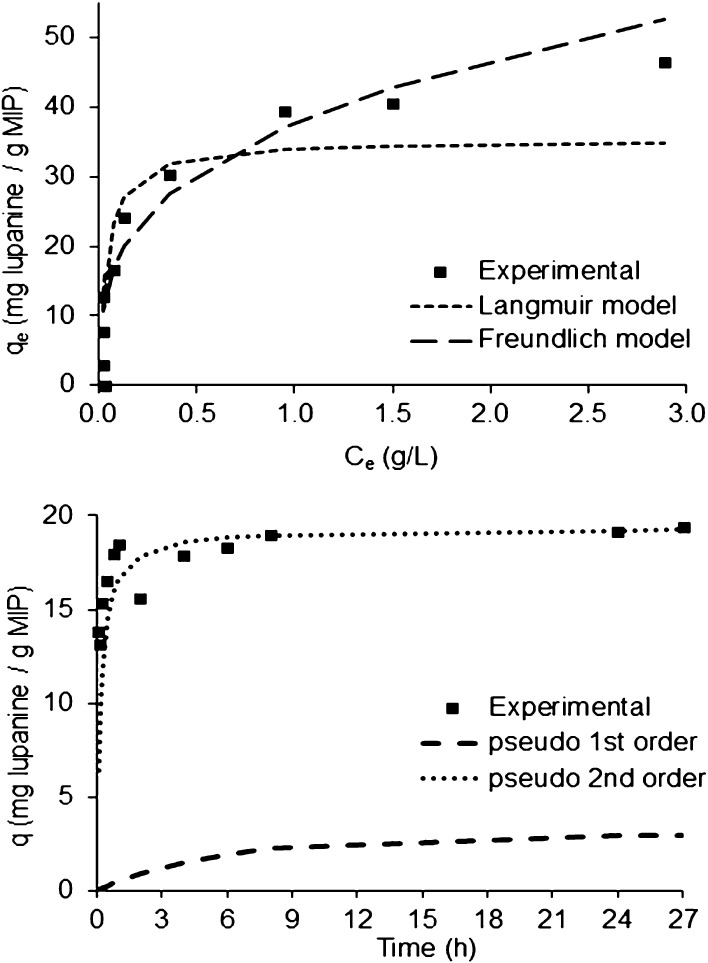
Isotherm adsorption (top) and kinetic models (bottom) for lupanine on **MIP-1** at room temperature in EtOAc.

The adsorption isotherm for lupanine on **MIP-1** is shown in Figure 7. The correlation coefficient for the Freundlich model (0.9323) is similar to the one of the Langmuir model (0.9302) (Table S3). However, the adjustment of the experimental data to both models shows a clear trend for the Freundlich isotherm, which considers that as lupanine concentration increases, the concentration of the alkaloid on the **MIP-1** surface will also increase.^65^ Furthermore, this model is typical of MIPs obtained by bulk polymerization, where grounding and sieving result in a heterogeneous binding site distribution.^60,61,66^ The binding kinetics was also assessed, showing that the binding process reaches equilibrium within 1 h, following a pseudo-second-order model (Figure 7 and Table S4).

#### 3.3.3 Recyclability and Lupanine Recovery

**Figure 8:**
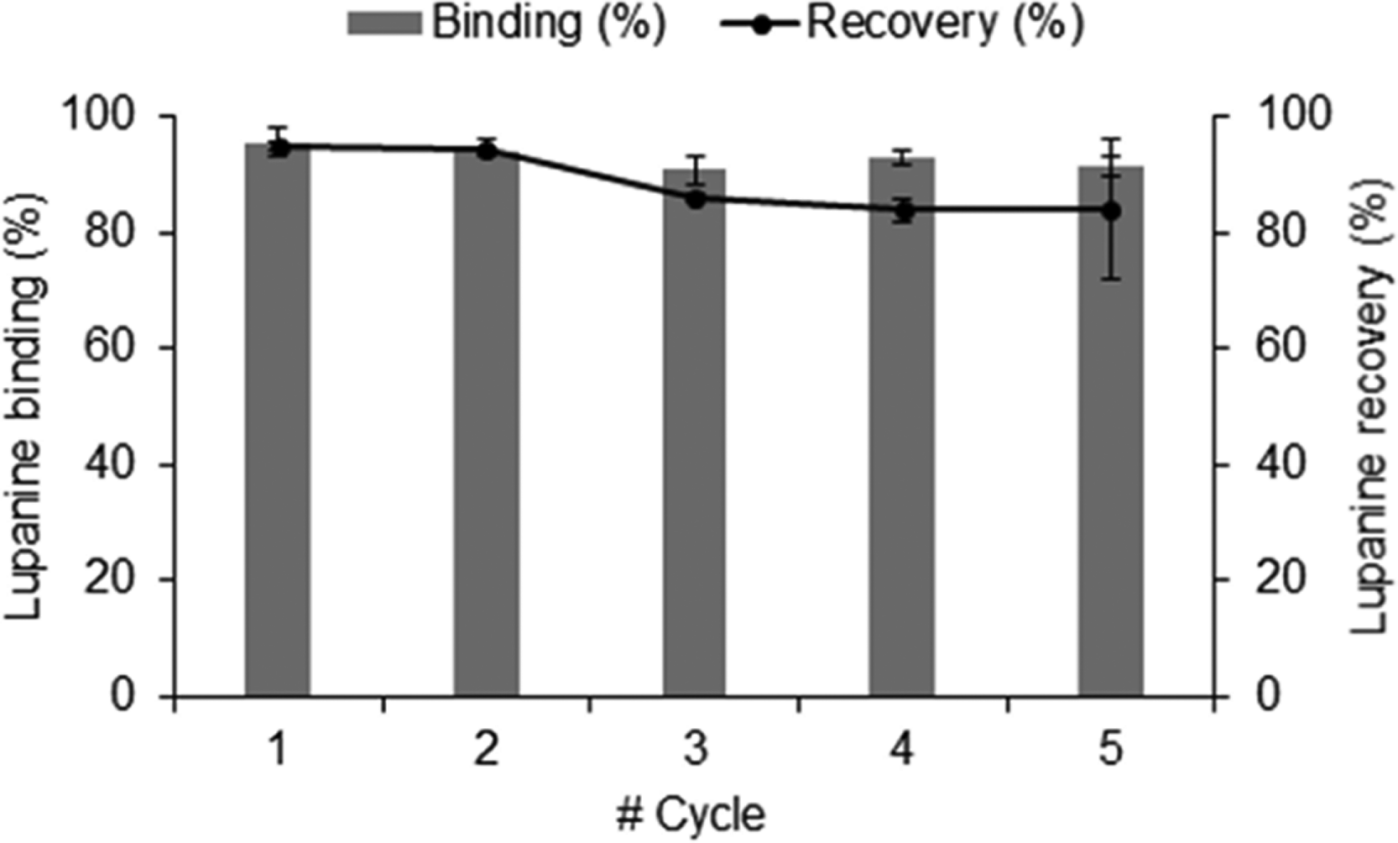
Cumulative recovery of lupanine in five consecutive binding/recovery cycles on **MIP-1** (*n* = 2).

The recovery of lupanine bound to the polymer is an important stage to be addressed in the overall process of isolation of this alkaloid from lupin bean wastewater. A solution of 0.1 M HCl in MeOH was used to recover lupanine from **MIP-1**. High polarity of MeOH enables the disruption of H bonds formed between the template and the functional monomer, while the presence of HCl promotes the protonation of carboxylic groups of IA and formation of ammonium chloride salt of lupanine. From Figure 8 it is possible to observe that **MIP-1** can be reused for at least five consecutive cycles of binding/recovery of lupanine with only around 25% loss in efficiency. A higher error was observed for the fifth cycle recovery, which may be due to sample handling, resulting in different **MIP-1** loss.

A possible strategy to facilitate the separation of the adsorber from the solution, after lupanine adsorption, would be the preparation of **MIP-1** on the surface of magnetic particles, minimizing sample handling. However, this strategy falls outside the scope of this paper but could be envisaged as an optimization of the proposed strategy.

### 3.4 Lupanine Isolation from Lupin Bean Industrial Wastewaters

As mentioned previously, we were able to isolate lupanine by SE with EtOAc from a lupin bean wastewater stream concentrated by NF.^18^ Here, we aim to find a strategy to improve even further the purity of lupanine that is recovered. Therefore, following the previously published procedure,^18^ from six consecutive lupin bean wastewater solvent-extracted with EtOAc, we obtained a lupanine-rich organic phase.

**Figure 9:**
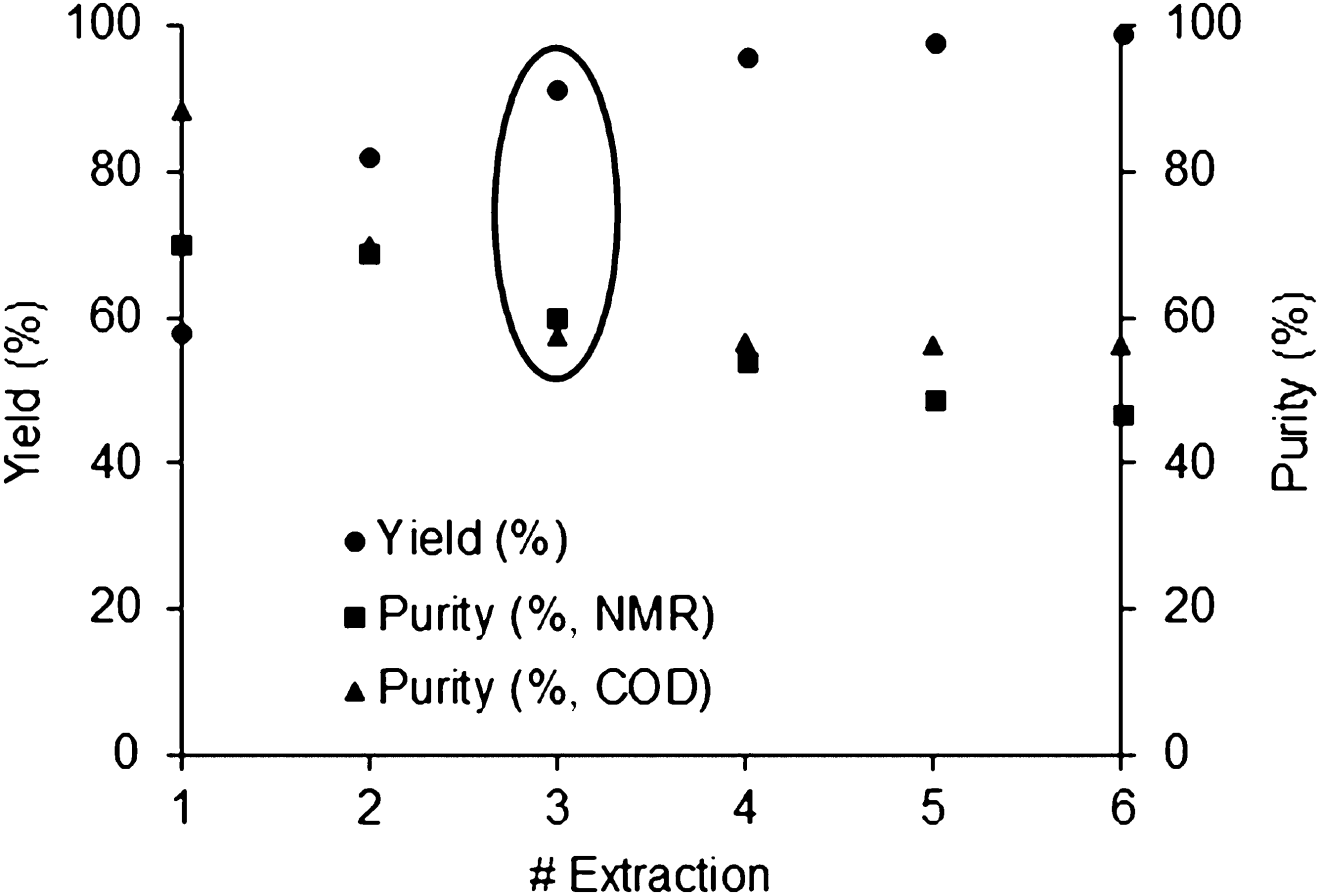
Performance of six multistep SE of lupin bean wastewater, with EtOAc, concerning lupanine recovery yield and purity.

From the results presented in Figure 9, at the third extraction step, we reached a lupanine yield and purity of 91% and (57–60)%, respectively. No further improvement in yield or purity is observed in the following SE steps. The difference between these results and the ones obtained previously by our group (98% yield and 78% purity, after three extractions)^18^ can be attributed to variation from batch to batch of processed dry lupin beans.

The organic solvent-extracting phase of the first three extractions was combined, resulting in an EtOAc solution with a lupanine concentration around 1.23 g/L. The performance of **MIP-1** in lupanine isolation from such solution was assessed performing the same protocol previously applied for pure lupanine samples. **MIP-1** was able to bind 98.5%, and 82.1% lupanine was recovered with around 88% purity. Remarkably, even in the presence of interfering molecules, possibly extracted from the wastewater together with lupanine into the EtOAc fraction, **MIP-1** kept its high performance (for pure lupanine samples, 92.9% binding and 87.3% recovery). However, some of these unknown compounds, although not competing with lupanine for the available binding sites, seem to be eluted together with lupanine during the recovery stage, and therefore, a purity of 88% was reached. Overall, **MIP-1** showed good performance for lupanine binding, comparable to the one obtained for pure lupanine samples in EtOAc, even in the presence of other unknown compounds.

#### 3.4.1 Process Design for Lupanine Isolation

We have previously reported a strategy to isolate lupanine from lupin bean wastewater using a NF stage, to concentrate the debittering wastewater, followed by SE with EtOAc of the lupanine-rich retentate obtained (NF + SE, Table 3).^18^ With this strategy, a recovery yield of around 95% was achieved with lupanine, presenting a final purity around 78%. In order to improve even further the purity of the crude obtained, in the current study, we assessed the use of **MIP-1** as a high-affinity adsorber for lupanine. In fact, by coupling the **MIP-1** adsorption stage to NF and SE (NF + SE + **MIP-1**), we were able to improve the purity of lupanine to around 88%, with an overall yield of 78% (Table 3). A lower overall yield is somehow expected as we are adding one more processing stage, and product losses occur during handling. When this strategy is compared with a process that comprises NF and SE, mediated by an adsorption stage using the commercial resin XAD-16 (NF + CC + SE, Table 3), the lupanine recovery yield is quite similar (74%). However, in this case, a lower-purity grade lupanine was obtained.

**Table 3:** Yield and Purity of Lupanine Isolated From Lupin Bean Wastewater^a^

	yield (%)	purity (%)
NF + SE^18^	95.4	78.4
NF + SE + **MIP-1**	78.3	88.0
NF + CC + SE^18^	74.4	78.4

a NF—nanofiltration, SE—solvent extraction, and CC—column chromatography with XAD-16 resin as a support.

When complex matrices are considered, such as the ones of wastewaters, NF, SE, or CC are separation techniques less selective than MIPs because their performance is based on differences on the molecular weight, ionization state, partition coefficient, or chemical functionalities between the target solutes and impurities. In our case study, the NF membrane presented a high rejection (95%) for lupanine and other organic species present in the wastewater.^18^ In SE, unknown compounds with a similar partition coefficient to the one of lupanine may be also extracted into the organic phase. Resin adsorption in CC relies on the retention of compounds with chemical functionalities similar to lupanine, which can also be co-eluted from the adsorber. Only the molecular imprinting provides the formation of binding sites on the adsorber, highly selective for lupanine, the target molecule used as template. Therefore, it was expected to achieve a higher lupanine purity based on this selectivity. However, some non-selective adsorption of other compounds present in the wastewater can also take place, contributing to the decrease in the final purity achieved.

**Figure 10:**
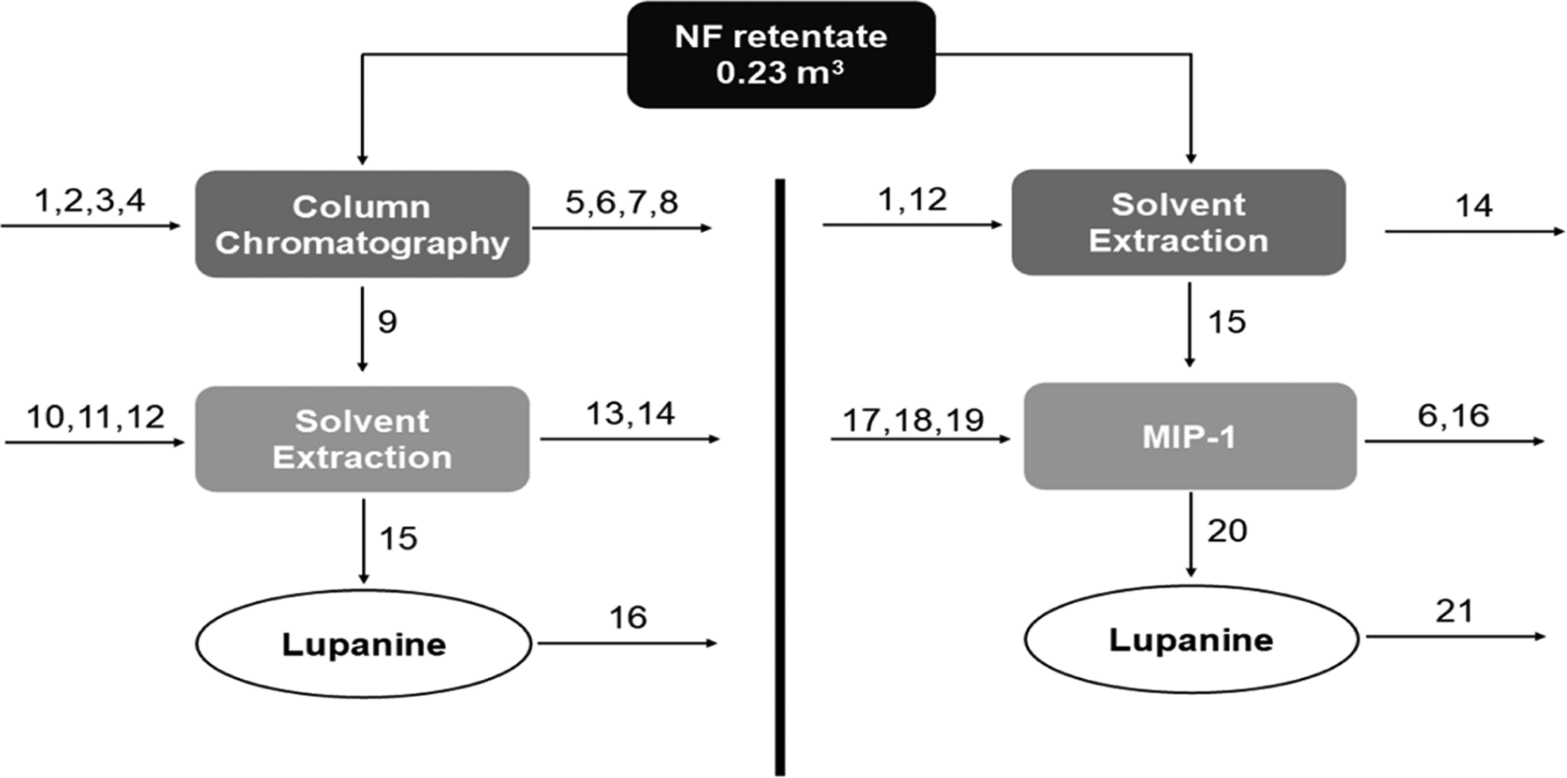
Proposed configurations for lupanine isolation from lupin bean wastewater.

Considering all the above, the strategy that includes the MIP (NF + SE + **MIP-1**, Figure 10, Table 4) is simpler than the one comprising XAD-16 resin column chromatography (CC) (NF + CC + SE). The lupanine is recovered from the XAD-16 resin using ethanol and submitted to a solvent swap with EtOAc before SE. In the presently proposed strategy, there is no need for solvent swap because **MIP-1** adsorption is fed directly with the lupanine-enriched organic solvent phase, obtained at the SE stage. The recovery of lupanine from **MIP-1** and its regeneration is less troublesome, using the same solvent (MeOH); whereas at the CC stage, ethanol and water are required for lupanine recovery and resin regeneration, respectively. Furthermore, during the **MIP-1** lupanine adsorption/recovery stages, no eluting fractions need to be monitored and collected through time, being less labor-intensive. However, in both strategies, the amount of the adsorber required was similar. There is to say that, for **MIP-1**, its performance in the dynamic mode was not assessed and probably could be improved due to the less adsorber being required to reach the same lupanine binding efficiency. The same reasoning can be applied for lupanine recovery from **MIP-1** because optimization of this stage was not fully investigated. These aspects could contribute to lowering the amount of the adsorber and recovery solvent needed to isolate lupanine.

**Table 4:** Comparison of Proposed Strategies for Lupanine Isolation From 1 m^3^ of Lupin Bean Wastewater Using **MIP-1** and a Commercial Resin (XAD-16) Coupled to NF, Based on Figure 10

stream	Description	NF + CC + SE^18^	NF + SE + **MIP-1**
1	NaOH (for pH adjustment)	11.5 Kg	11.5 Kg
2	adsorber (XAD-16)	34.5 Kg	
3	EtOH (for lupanine recovery from XAD-16)	0.14 m^3^	
4	aqueous stream for regeneration of XAD-16	0.14 m^3^	
5	spent basified water from column adsorption	0.23 m^3^	
6	spent adsorber	34.5 Kg	
7	spent aqueous stream from adsorber regeneration	0.14 m^3^	
8	spent EtOH from lupanine recovery	0.10 m^3^	
9	concentrated organic phase enriched in lupanine (EtOH)	0.034 m^3^	
10	water for dissolution of dry residue from EtOH	0.034 m^3^	
11	NaOH for aqueous phase pH adjustment	0.72 Kg	
12	organic solvent (EtOAc)	0.10 m^3^	0.69 m^3^
13	spent EtOH	0.034 m^3^	
14	spent aqueous phase	0.034 m^3^	0.23 m^3^
15	organic phase rich in lupanine (EtOAc)	0.10 m^3^	0.69 m^3^
16	spent EtOAc	0.10 m^3^	0.69 m^3^
17	adsorber (**MIP-1**)		34.5 Kg
18	HCl 0.1 M/MeOH (for lupanine recovery from **MIP-1**)		1.38 m^3^
19	MeOH (for **MIP-1** regeneration)		0.69 m^3^
20	HCl/MeOH fraction rich in lupanine		2.07 m^3^
21	spent HCl 0.1 M/MeOH (for lupanine recovery from **MIP-1**)		2.07 m^3^

Other methods are reported for the isolation of lupanine. A reverse osmosis stage coupled to solid–liquid extraction with ethyl ether resulted in only 18.5% isolation of lupanine from lupin bean wastewaters with around 90% purity.^17^ Although the authors achieved a higher final purity in such a study, the yield was very low compared to the one obtained with our proposed strategy. Furthermore, as previously observed by our group, conversion of lupanine, with 78% purity, to pure sparteine, was successfully achieved.^18^ Therefore, we proposed a polishing step with **MIP-1** delivering a higher amount of final product with an acceptable purity for further chemical transformation.

## 4 Conclusions

In this work, molecular modeling studies showed that the interaction between lupanine and IA was favored due to the presence of a more extensive non-covalent interaction surface combined with a more stable electrostatic component, for weak interactions, compared to the remaining functional monomers. Based on this, a molecularly imprinted polymer (**MIP-1**) was developed for selective binding of lupanine using IA (a bio-based building block) as a functional monomer and EGDMA as a cross-linker by bulk polymerization. A higher performance for lupanine recognition (>95%) than the respective **NIP-1** (<40%), in several organic solvents (EtOAc, MTBE, EtOH, and DCM), was obtained, despite a smaller surface area of **MIP-1** (37.53 m^2^/g) compared to **NIP-1** (271.23 m^2^/g), indicating that the use of this MIP for further lupanine purification from EtOAc, after solvent extraction, may be a strategy worth exploring. **MIP-1** showed an adsorption process following the Freundlich model, reaching equilibrium within 1 h by a pseudo-second-order kinetic model. **MIP-1** allowed a good recovery yield of lupanine (82–87%) both for pure lupanine samples and the ones driven from the industrial wastewater. By coupling the **MIP-1** adsorption stage to NF and SE (NF + SE + **MIP-1**), it was possible to recover lupanine with 88% purity. This result shows an improvement compared to previous strategies which, after the NF stage, use SE alone or include a XAD-16 resin adsorption stage, reaching a final lupanine purity of only 78%. These results show the potential applicability of a high-affinity material to isolate an added-value compound from waste generated from the food industry, rendering the process more sustainable.

## Supporting Information Available

Additional characterization details for **MIP-1** and **NIP-1** including FTIR-ATR spectra, histogram for particle size, imprinting factors, elemental analysis, equilibrium isotherm and kinetic model parameters, and HPLC chromatograms (PDF)
