# Supplementary material for: Greener Strategy for Lupanine Purification from Lupin Bean Wastewaters Using a Molecularly Imprinted Polymer
Source: ACS Appl Mater Interfaces. 2022 Apr 14;14(16):18910–21. doi: 10.1021/acsami.2c02053 (PMC9773177; doi:10.1021/acsami.2c02053)
Supplement: Supplementary file 1 — am2c02053_si_001.pdf [file am2c02053_si_001.pdf]

# Supporting Information

# Greener strategy for lupanine purification from lupin beans wastewaters using a molecularly imprinted polymer

Teresa Esteves[a,b*], Flávio A. Ferreira[a,b], Ana Teresa Mota[a,b], Ángel Sánchez-González[c], Adrià Gil[c*], Késsia H. S. Andrade[d], Carlos A. M. Afonso[d], Frederico Castelo Ferreira[a,b]

[a] iBB - Institute for Bioengineering and Biosciences and Department of Bioengineering, Instituto Superior Técnico, Universidade de Lisboa, Av. Rovisco Pais, 1049-001 Lisboa, Portugal.
[b] Associate Laboratory i4HB—Institute for Health and Bioeconomy at Instituto Superior Técnico, Universidade de Lisboa, Av. Rovisco Pais, 1049-001 Lisboa, Portugal.
[c] Centro de Química e Bioquímica and BioISI – Biosystems and Integrative Sciences Institute, DQB, Faculdade de Ciências, Universidade de Lisboa, Campo Grande, 1749-016 Lisboa, Portugal.
[d] Research Institute for Medicine (iMED, ULisboa); Faculty of Pharmacy, Universidade de Lisboa, Avenida Prof. Gama Pinto, 1649-003 Lisboa, Portugal.

**Corresponding author:**
*Teresa Sofia Araújo Esteves; iBB - Institute for Bioengineering and Biosciences and Department of Bioengineering, Instituto Superior Técnico, Universidade de Lisboa, Av. Rovisco Pais, 1049-001 Lisboa, Portugal. Associate Laboratory i4HB—Institute for Health and Bioeconomy at Instituto Superior Técnico, Universidade de Lisboa, Av. Rovisco Pais, 1049-001 Lisboa, Portugal. Tel: +351 218419167; teresa.esteves@tecnico.ulisboa.pt;
*Adriá Gil Mestres; Centro de Química e Bioquímica and BioISI – Biosystems and Integrative Sciences Institute, DQB, Faculdade de Ciências, Universidade de Lisboa, Campo Grande, 1749-016 Lisboa, Portugal; agmestres@fc.ul.pt.

## Table of Contents

**Figure S1.** HPLC chromatogram of pure lupanine ............................... S3
**Figure S2.** HPLC chromatogram of lupin beans wastewater ................... S3
**Figure S3.** HPLC chromatogram from lupanine fraction recovered from **MIP-1** ........................................................................................ S3
**Figure S4.** FTIR-ATR spectra of **MIP-1**, **NIP-1**, lupanine and IA ............ S4
**Figure S5.** Histogram for particle size .............................................. S4
**Table S1.** Imprinting factor for polymers .......................................... S5
**Table S2.** Elemental analysis ............................................................ S5
**Table S3.** Equilibrium isotherm model parameters ............................. S5
**Table S4.** Kinetic model parameters ................................................ S5

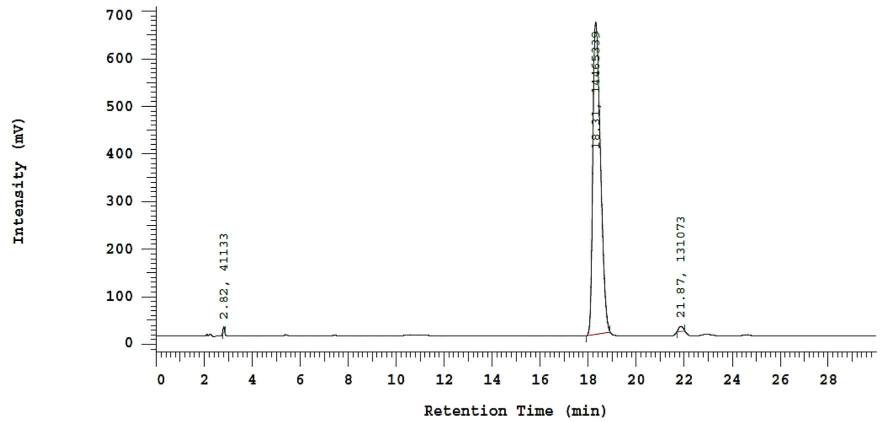


**Figure S1.** HPLC chromatogram of lupanine pure sample prepared in water.

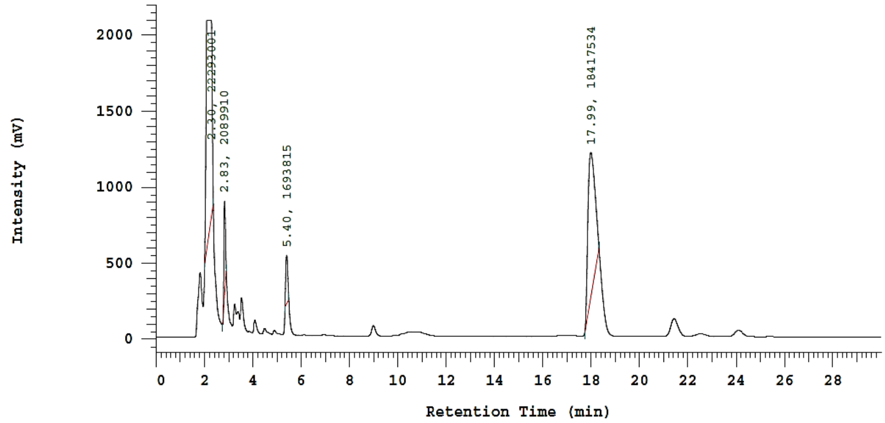


**Figure S2.** HPLC chromatogram of lupin beans wastewater after pH adjustment to 13-13.5 with KOH pellets.

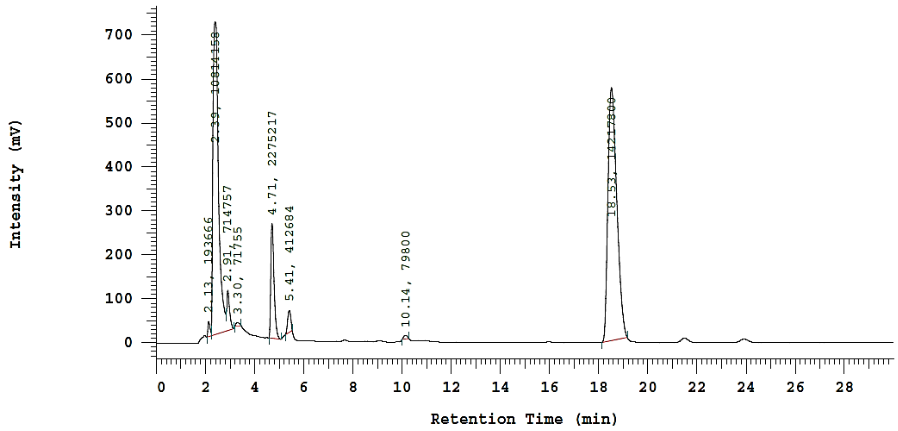


**Figure S3.** HPLC chromatogram of lupanine recovered from **MIP-1** after MeOH washing, solvent evaporation, dissolution in water and pH adjustment to 13-13.5 with KOH pellets.

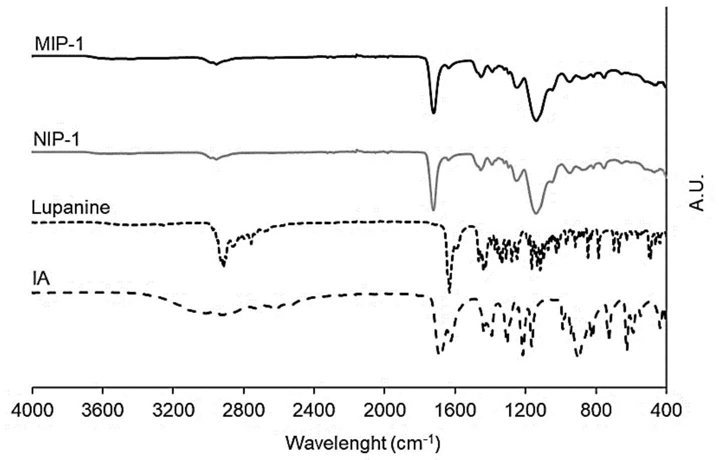


**Figure S4.** FTIR-ATR spectra of **MIP-1**, **NIP-1**, lupanine and IA.

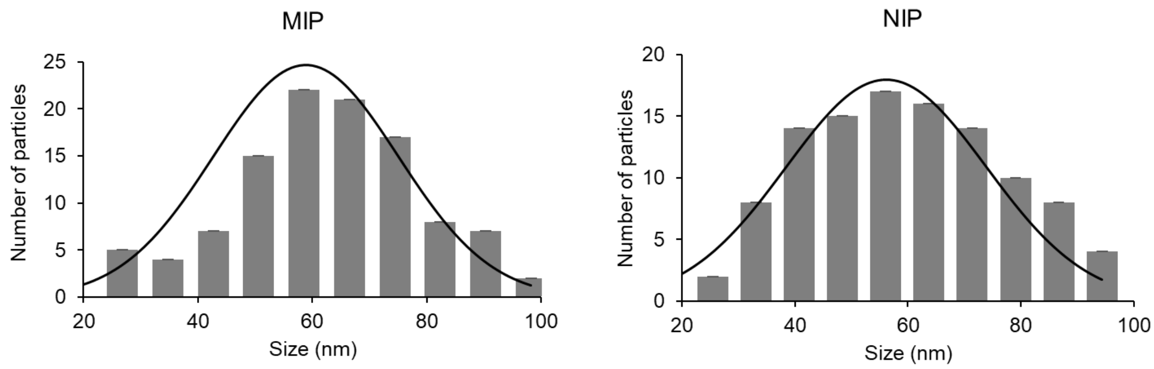


**Figure S5.** Histogram of the diameter distribution for **MIP-1** (left) and **NIP-1** (right) particles.

**Table S1.** Imprinting factors calculate for the several MIPs prepared in different solvents for lupanine solutions prepared at 1 g/L.

| Functional Monomer | Solvent DCM | MTBE | EtOAc | EtOH |
|---|---|---|---|---|
| **IA** | 1.5 | 3.7 | 2.3 | 2.7 |
| **MAA** | 1.8 | 2.1 | 2.0 | 1.3 |
| **MMA** | 11.0 | 0.7 | 0.1 | 11.0 |
| **NIPAM** | 6.4 | 0.7 | 0.1 | 1.1 |
| **Styrene** | 1.2 | 1.1 | 1.5 | 1.0 |

**Table S2.** Elemental analysis for **MIP-1** and **NIP-1**.

| | | Found (%) | |
|---|---|---|---|
| **Element** | **Calcd. (%)** | **MIP-1** | **NIP-1** |
| C | 58.91 | 57.33 | 58.16 |
| H | 6.83 | 7.20 | 7.26 |
| N | - | - | - |

**Table S3.** Equilibrium isotherm model parameters for lupanine on **MIP-1** at room temperature in EtOAc.

| Parameters | | | | | |
|---|---|---|---|---|---|
| Langmuir | | | Freundlich | | |
| $q_m$ (g/g) | $K_L$ (L/g) | $R^2$ | n | $K_F$ ((g/g)(L/g)$^{1/n}$) | $R^2$ |
| 0.035 | 25.26 | 0.9302 | 3.197 | 0.04 | 0.9323 |

**Table S4.** Kinetic parameters for lupanine on **MIP-1** at room temperature in EtOAc.

| Pseudo-first order | | | Pseudo-second order | | |
|---|---|---|---|---|---|
| $k_1$ ($h^{-1}$) | $q_e$ (mg/g) | $R^2$ | $k_2$ ((g/(mg·h)) | $q_e$ (mg/g) | $R^2$ |
| 0.18 | 2.96 | 0.7618 | 0.31 | 19.37 | 0.9997 |
